# Invasiveness and surgical timing evaluation by clinical features of ground‐glass opacity nodules in lung cancers

**DOI:** 10.1111/1759-7714.13199

**Published:** 2019-09-30

**Authors:** Pai‐Hsi Chen, Kuo‐Ming Chang, Wei‐Chi Tseng, Chien‐Hung Chen, Jui‐I Chao

**Affiliations:** ^1^ Department of Surgery Hsinchu Mackay Memorial Hospital Hsinchu Taiwan; ^2^ Department and Institute of Biological Science and Technology National Chiao Tung University Hsinchu Taiwan; ^3^ Department of Pathology Hsinchu Mackay Memorial Hospital Hsinchu Taiwan; ^4^ Department of Radiology Hsinchu Mackay Memorial Hospital Hsinchu Taiwan; ^5^ Institute of Molecular Medicine and Bioengineering National Chiao Tung University Hsinchu Taiwan; ^6^ Center For Intelligent Drug Systems and Smart Bio‐devices National Chiao Tung University Hsinchu Taiwan

**Keywords:** Ground‐glass opacity, invasiveness, lung adenocarcinoma, surgical intervention

## Abstract

**Background:**

The early stages of lung cancer with ground‐glass opacity (GGO) pattern are detectable. However, it remains a challenge for physicians how best to treat GGO nodules as invasive tumors are occasionally found, even in pure GGO nodules. This study identified the invasiveness by the clinical features of the GGO nodules.

**Methods:**

A retrospective review of patients with resected GGO nodules from August 2015 to February 2019 was performed. A total of 92 patients were enrolled and gender, age, tumor location, operation times, tumor size, histopathologic and radiological findings were analyzed.

**Results:**

In this study, the sequential of GGO nodules invasiveness was significantly related to the tumor size and solid component. After regrouping the population into preinvasive and invasive groups, the invasiveness was significantly related to tumor size, solid component, tumor volume and maximal computed tomography (CT) value.

**Conclusions:**

The invasiveness is difficult to evaluate according to the CT features only when the GGO nodules are less than 2 cm and consolidation/tumor ratio (C/T ratio) are less than 0.25. Tumor size and solid component are significant factors for predicting invasiveness. Part‐solid GGO nodules with a diameter greater than 1 cm require surgical consideration due to their high risk of invasiveness.

## Key points

**Significant findings of the study**: Tumor size and solid component are significant factors for predicting invasiveness. In this study, the sequential development of GGO nodule invasiveness was significantly related to the tumor size and solid component.

**What this study adds**: This study determined that part‐solid GGO nodules with a diameter larger than 1 cm should be considered for surgery due to their high risk of invasiveness.

## Introduction

Lung cancer is one of the most prevalent lethal diseases in the world,^1^ and with the increased use of computed tomography (CT) scanning for lung cancer screening, ground‐glass opacity (GGO) nodules are encountered more frequently.^2^ The GGO nodules result from a variety of differential diagnosis of benign and malignant diseases including inflammatory process, focal fibrosis, atypical adenomatous hyperplasia (AAH), adenocarcinoma in situ (AIS), minimally invasive adenocarcinoma (MIA), and invasive adenocarcinoma (IA).^3^ For clinical physicians, it is a challenge to diagnose GGO nodules using the features of the CT images and thus results in an important issue on how best to manage the GGO nodules.

According to the National Comprehensive Cancer Network (NCCN) guidelines for lung cancer screening (Version 2.2019), pure GGO or part‐solid GGO with a solid component less than 5 mm should be closely followed‐up. For patients with AIS or MIA presenting with GGO nodules, an annual follow‐up CT scan may be reasonable and safe due to its 100% five‐year survival rate.^4^, ^5^ In patients diagnosed with IA, the five‐year survival rate is only 74.6%^6^ and surgical intervention as early as possible is critical. However, IA has been reported in the pure GGO or part‐solid GGO nodules with a solid component less than 5 mm,^7^, ^8^ which is determined as radiologic AIS or MIA, and that brings about an unsatisfactory outcome if the clinical physicians do not closely follow‐up IA patients.

Surgical timing is crucial for the GGO nodules, especially when invasiveness occurs. However, the optimal timing of surgical intervention for the GGO nodules is debatable due to their uncertainty.^9^, ^10^ The aim of this study was to identify the invasiveness of the GGO nodules by the clinical features of the CT scan and offer a valuable guide for clinical physicians to manage GGO nodules.

## Methods

We performed a retrospective record and pathologic review from August 2015 to February 2019 at the Hsinchu Mackay Memorial Hospital, Taiwan. The study was approved by the Mackay Memorial Hospital Institutional Ethics Committee/Review Board (18MMHIS129).

## Study population and design

From August 2015, patients with GGO nodules with a maximum diameter of 2 cm or less and consolidation/tumor ratio (C/T ratio) less than 0.25 were enrolled into the study. Through February 2019, we collected information on 99 patients who were considered to have clinically early stage lung cancer with pure or part‐solid GGO nodules which had been surgically removed. Among these patients, a total of seven were excluded. Six patients had synchronous lesions and one patient had a large mediastinal tumor which was resected during the same operation. A total of 92 patients were finally enrolled into the study. Medical records containing patient characteristics, features of GGO nodules, operative procedures, locations of the lesions, operative times, pathologic results, and radiologic findings were reviewed and analyzed.

## Image analysis

All the images were taken using the multidetector CT system: Somatom Definition AS+ (Siemens Medical Solutions, Erlangen, Germany) with a section thickness of 1 mm using the lung window setting (window level, −500 HU; width, 1500 HU). The images were evaluated by five experienced radiologists. The pure GGO nodule was defined as a homogeneous hazy tumor that did not obscure the underlying structures or vessels, and the part‐solid GGO nodule was defined as a lesion which had solid component in a homogeneous background with increasing attenuation. The tumor size was measured in the maximal cross‐section diameter on the lung window setting.

The tumor volume and attenuation were analyzed by one radiological technologist who was unaware of the clinical and pathologic reports of the patients. Prior to calculation, the tumor was marginated by drawing freehand a circular line with the computer mouse to cover an area as large as the whole tumor. The tumor volume, maximal diameter, and attenuation could then be calculated using Vitrea Enterprise Suite software (version 6.8).

## Surgical procedure

Tumor localization was the first step for the patients who received the surgical resection. The patients were first referred to the radiological department for the CT‐guided localization with patent blue V dye (PBV 2.5%; Guerbet, Aulnay‐sous‐Bois, France) injection. The surgical procedures included wedge resection, segmentectomy, and lobectomy, which were chosen according to the tumor sizes, locations, and their characteristics. All surgical procedures were performed with single‐incisional video‐assisted thoracic surgery (VATS) by one thoracic surgeon.

## Pathologic classification

All the specimens were examined by three experienced pathologists. The GGO nodule was classified as AAH, AIS, MIA, and IA according to the 2015 World Health Organization (WHO) classification of lung tumors and the final pathologic reports were reconfirmed by the other pathologist.

## Statistical analysis

The Pearson chi‐square test or Fisher's exact test was used to compare categorical variables between groups. The statistical analysis of continuous variables was performed by Student's *t‐*test and one‐way analysis of variance (ANOVA). A two‐tailed *P*‐value of <0.05 was considered statistically significant. Analyses were carried out using the statistical package SPSS 17.0 (IBM, Armonk, New York, USA).

## Results

### Baseline characteristics

**Figure 1:**
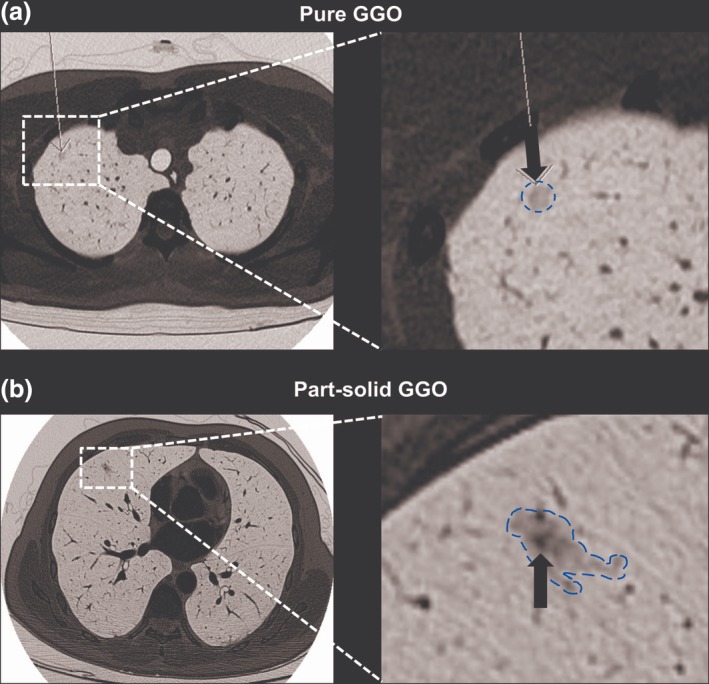
Ground‐glass opacity (GGO) nodules diagnosed by chest CT scan. (a) Pure GGO nodule. One 0.5 x 0.5 cm tiny GGO lesion was detected. Focusing on the GGO lesion, there was no solid component (arrow). Atypical adenomatous hyperplasia (AAH) was the definitive pathological diagnosis following resection. (b) Part‐solid GGO nodule. One 1.1 x 0.6 cm tiny GGO lesion was noted in the right middle lobe of lung. A solid component (arrow) in the GGO lesion was also noted. The final diagnosis after resection revealed minimally invasive adenocarcinoma (MIA).

All 92 patients were classified into two groups: 59 patients in the pure GGO group and 33 patients in the part‐solid GGO group. The images of pure GGO nodules and part‐solid GGO nodules are shown in Fig 1a,b, respectively. All the consolidation/tumor ratios (C/T ratios) of part‐solid GGO nodules included in our study were less than 0.25. The clinical parameters and the features are summarized in Table 1. The characteristics of the patients were analyzed and there were no significant difference of gender, age, surgical procedure, and operative times between pure GGO and part‐solid GGO group. The mean tumor size was 0.78 cm and 1.04 cm in the pure GGO group and the part‐solid GGO group, respectively. The mean tumor size in the part‐solid GGO group was significantly greater than those in the pure GGO group (*P* = 0.003). In the histopathologic results, we found that there were 25 (42.4%) AAH, 32 (54.2%) AIS, and two (3.4%) MIA who presented with pure GGO nodules; in contrast, there were seven (21.2%) AAH, 18 (54.5%) AIS, seven (21.2%) MIA, and one (3.0%) IA who presented with part‐solid GGO nodules. The distribution of the adenocarcinoma subtypes showed significant difference in invasiveness between the pure GGO and part‐solid GGO group (*P* = 0.01).

**Table 1:** Comparison of the pure GGO and part‐solid GGO nodules with different characteristics

Parameters	Pure GGO (*n* = 59)	Part‐solid GGO (*n* = 33)	*P*‐value
Gender, *n* (%)			
Male	21 (35.6)	17 (51.5)	0.137
Female	38 (64.4)	16 (48.5)	
Age (years), median (range)	49 (28–72)	54 (33–88)	0.173
Operation, *n* (%)			
Wedge resection	48 (81.4)	25 (75.8)	0.725
Segmentectomy	8 (13.6)	5 (15.2)	
Lobectomy	3 (5.1)	3 (9.1)	
Operative times (minute), mean (range)			
Wedge resection	65 (29–129)	57 (21–83)	0.093
Segmentectomy	154 (76–265)	199 (145–290)	0.218
Lobectomy	228 (164–281)	278 (245–323)	0.300
Tumor size (cm), mean (range)	0.78 (0.4–1.8)	1.04 (0.3–1.9)	0.003
Histopathology, *n* (%)			
AAH	25 (42.4)	7 (21.2)	0.010
AIS	32 (54.2)	18 (54.5)	
MIA	2 (3.4)	7 (21.2)	
IA	0 (0)	1 (3.0)	

GGO, ground‐glass opacity; AAH, atypical adenomatous hyperplasia; AIS, adenocarcinoma in situ; MIA, minimally invasive adenocarcinoma; IA, invasive adenocarcinoma.

### Characteristics of the different histopathologic subtypes

**Figure 2:**
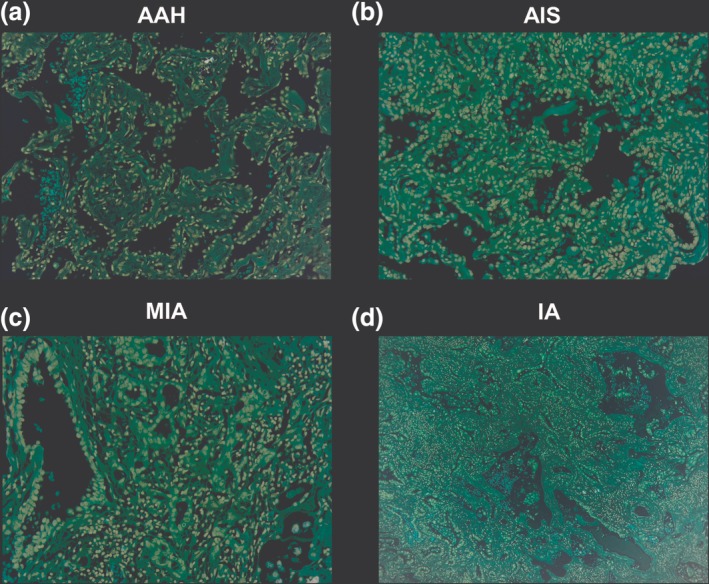
Demonstration of the histopathologic images of the early lung adenocarcinoma subtypes. (**a**) Atypical adenomatous hyperplasia (AAH). The tumor cells show a purely lepidic pattern without stoma invasion (150x); (**c**) Minimally invasive adenocarcinoma (MIA). Note that the tumor consists of lepidic growth with a small central area of invasion <0.5 cm (150x); (**d**) IA: Lepidic growth of the tumor with central desmoid reaction is visible. The invasive area >0.5 cm in diameter (150x).

**Figure 3:**
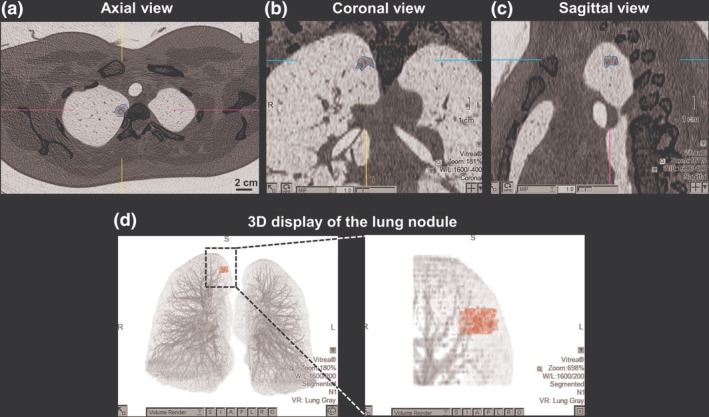
The tumor volume and value of the ground‐glass opacity (GGO) nodule were analyzed. The tumor was marginated by drawing freehand a circular line with the computer mouse and the scale was calculated with Vitrea Enterprise Suite software. (**a**) Axial view of the CT scan. A yellowish circular line was drawn by the radiological technologist. (**b**) & (**c**) Coronal and sagittal views of the CT scan. The radiological technologist adjusted the circular line precisely to the margin of the tumor. (**d**) Three‐dimensional display of the lung nodule. According to the graphic range marginated in axial, coronal, and sagittal view, the nodule was presented automatically by the software. The tumor volume and value were calculated and displayed.

The histopathologic subtypes of the GGO nodules were diagnosed and the histopathologic images of AAH (Fig 2a), AIS (Fig 2b), MIA (Fig 2c), and IA (Fig 2d) were shown. Moreover, the tumor volume and attenuation values of the GGO nodules, which were calculated using Vitrea Enterprise Suite software were selected for invasiveness evaluation (Fig 3). As detailed in Table 2, the patient's numbers of four subtypes were 32 patients in AAH (34.8%), 50 in AIS (54.3%), nine in MIA (9.8%), and one patient in IA (1.1%). We evaluated the gender, age, tumor size, tumor location, and CT findings in these four histopathologic subtypes. A significant difference was noted in the average tumor size (0.74 cm in AAH, 0.88 cm in AIS, 1.20 cm in MIA and 1.9 cm in IA, *P* = 0.009).

**Table 2:** Comparison of different histopathologic subtypes of early lung adenocarcinomas

Parameters	AAH (*n* = 32)	AIS (*n* = 50)	MIA (*n* = 9)	IA (*n* = 1)	*P*‐value
Gender, *n* (%)					
Male	9 (28.1)	22 (44.0)	6 (66.7)	1 (100)	0.100
Female	23 (71.9)	28 (56.0)	3 (33.3)	0 (0)	
Age (years), median (range)	49 (31–70)	50 (28–72)	62 (35–88)	59	0.220
Tumor size (cm), mean (range)	0.74 (0.3–1.5)	0.88 (0.5–1.8)	1.20 (0.5–1.6)	1.9 (1.9)	<0.001
Location, *n* (%)					
Right upper lobe	15 (46.9)	25 (50.0)	1 (11.1)	1 (100)	0.489
Right middle lobe	3 (9.4)	3 (6.0)	0 (0)	0 (0)	
Right lower lobe	3 (9.4)	7 (14.0)	4 (44.4)	0 (0)	
Left upper lobe	7 (21.9)	11 (22.0)	3 (33.3)	0 (0)	
Left lower lobe	4 (12.5)	4 (8.0)	1 (11.1)	0 (0)	
CT findings					
Pure GGO lesion, *n* (%)	25 (78.1)	32 (64.0)	2 (22.2)	0 (0)	0.010
Part‐solid GGO lesion, *n* (%)	7 (21.9)	18 (36.0)	7 (77.8)	1 (100)	
Tumor volume (mm^3^), mean ± SD	79 ± 92	199 ± 278	442 ± 351	321.9	0.001
Mean tumor attenuation (HU), mean ± SD	−531 ± 164	−549 ± 133	−485 ± 132	−356	0.390
Maximum tumor attenuation (HU), mean ± SD	−162 ± 219	−121 ± 238	69 ± 158	6	0.058
Minimum tumor attenuation (HU), mean ± SD	−864 ± 149	−887 ± 118	−840 ± 92	−650	0.236

AAH, atypical adenomatous hyperplasia; AIS, adenocarcinoma in situ; MIA, minimally invasive adenocarcinoma; IA, invasive adenocarcinoma; Preinvasive, AAH + AIS; Invasive, MIA + IA; GGO, ground‐glass opacity.

With regard to the results of CT findings, we found a trend that the percentage of part‐solid GGO lesions and mean tumor volume significantly increased with the progression of the invasiveness (the percentage of part‐solid GGO lesions: 21.9% in AAH, 36.0% in AIS, 77.8% in MIA, and 100% in IA, *P* = 0.01; mean tumor volume: 79 mm^3^ in AAH, 199 mm^3^ in AIS, 442 mm^3^ in MIA, and 321.9 mm^3^ in IA, *P* = 0.001).

### Characteristics of invasiveness

To investigate invasiveness of the GGO lesions, we grouped AAH and AIS into the preinvasive group and grouped MIA and IA into the invasive group, as shown in Table 3. There were no significant differences in gender, age, and location. Tumor sizes in the invasive group were significantly larger than those in preinvasive group (1.27 cm vs. 0.83 cm, *P* < 0.001). The CT findings were evaluated and preinvasive nodules were more likely to present in a pure GGO pattern, whereas the pure GGO pattern was only present in 20% of invasive nodules (*P* = 0.004). The tumor volume was also significantly different between the preinvasive and invasive groups (*P* = 0.001). Furthermore, maximal tumor attenuation revealed a significant difference between the preinvasive and invasive group (*P* = 0.002). After the cutoff value of tumor size was set at 1 cm, the results showed that in the pure GGO group there was no significant difference between tumor size and invasiveness; on the contrary, a nodule larger than 1 cm was a significant factor to predict invasiveness in the part‐solid group (*P* = 0.012, OR = 14.88) (Table 4).

**Table 3:** Relationships between the clinical characteristics and histopathologic invasiveness

Parameters	Preinvasive (*n* = 82)	Invasive (*n* = 10)	*P*‐value
Gender, *n* (%)			
Male	31 (37.8)	7 (70)	0.086
Female	51 (62.2)	3 (30)	
Age (years), median (range)	50 (28–72)	61 (35–88)	0.169
Tumor size (cm), mean (range)	0.83 (0.3–1.8)	1.27 (0.5–1.9)	<0.001
Location, *n* (%)			
Right upper lobe	40 (48.8)	2 (20.0)	0.129
Right middle lobe	6 (7.3)	0 (0)	
Right lower lobe	10 (12.2)	4 (40.0)	
Left upper lobe	18 (22.0)	3 (30.0)	
Left lower lobe	8 (9.8)	1 (10.0)	
CT findings			
pure GGO lesion, *n* (%)	57 (69.5)	2 (20.0)	0.004†
part‐solid GGO lesion, *n* (%)	25 (30.5)	8 (80.0)	
Tumor volume (mm^3^), mean ± SD	152 ± 231	430 ± 333	0.001
Mean tumor attenuation (HU), mean ± SD	−541 ± 145	−472 ± 131	0.147
Maximum tumor attenuation (HU), mean ± SD	−137 ± 230	63 ± 151	0.002
Minimum tumor attenuation (HU), mean ± SD	−876 ± 131	−821 ± 106	0.193

Preinvasive, AAH + AIS; Invasive, MIA + IA.

† Odds ratio = 9.12, 95% Confidence interval = 1.81–46.05.

**Table 4:** Relationship between invasiveness and tumor size in different GGO groups

	Preinvasive	Invasive	OR (95% C.I.)	*P*‐value
Pure GGO, *n* (%)				
**≤** 1 cm	51 (89.5)	2 (100)	1	
> 1 cm	6 (10.5)	0 (0)	0.96 (0.91–1.02)	1
Part‐solid GGO, *n* (%)				
**≤** 1 cm	17 (68.0)	1 (12.5)	1	
> 1 cm	8 (32.0)	7 (87.5)	14.88 (1.56–142.20)	0.012

Preinvasive, AAH + AIS; Invasive, MIA + IA; OR, odds ratio; C.I., confidence interval; GGO, ground‐glass opacity.

### Correlation between GGO nodule morphology and histopathologic invasiveness

**Figure 4:**
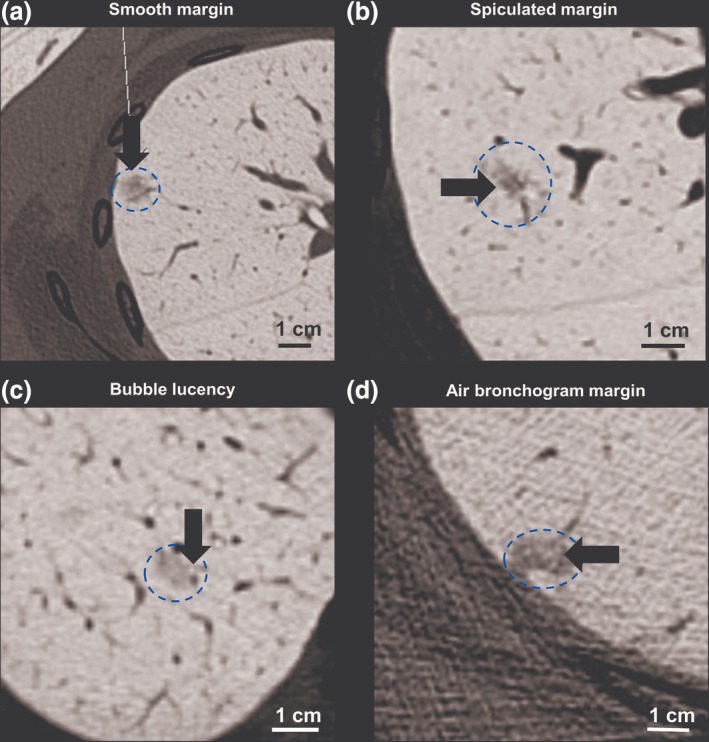
Morphology of the ground‐glass GGO nodules visible on the CT scan. (**a**) A smooth margin (arrow) of one pure GGO nodule was noted in a 50‐year‐old female with a final pathologic report of adenocarcinoma in situ (AIS). (**b**) Spiculated margin (arrow) of part‐solid GGO nodule. The final histopathology was minimally invasive adenocarcinoma (MIA). (**c**) Bubble lucency (arrow) in a pure GGO nodule. This image was from a 51‐year‐old female with AIS. (**d**) Air bronchogram (arrow). There is one V‐shape air‐filled bronchus visible in a GGO nodule which post‐operatively was diagnosed as MIA.

Except for the CT attenuation value, we analyzed the GGO nodule morphology for invasiveness in the pure GGO group and part‐solid GGO group. The nodular margin, bubble lucency, and air bronchogram (Fig 4) were analyzed in the different groups, respectively. All these three features revealed no significant difference between preinvasive and invasive nodules in both pure GGO, or part‐solid GGO groups. According to this result, morphology of the GGO nodule could not be considered as the predictor for the invasiveness evaluation (Table 5).

**Table 5:** Comparison of the relationship between the invasiveness and GGO morphology in pure GGO and part‐solid GGO groups

	Pure GGO (*n* = 59)			Part‐solid GGO (*n* = 33)		
Parameters	Preinvasive (*n* = 57)	Invasive (*n* = 2)	*P*‐value	Preinvasive (*n* = 25)	Invasive (*n* = 8)	*P*‐value
Margin, *n* (%)						
Smooth	43 (75.4)	1 (50.0)	0.447	9 (36.0)	1 (12.5)	0.382
Spiculated	14 (24.6)	1 (50.0)		16 (64.0)	7 (87.5)	
Bubble lucency sign, *n* (%)						
Yes	29 (50.9)	0 (0.0)	0.492	10 (40.0)	2 (25.0)	0.678
No	28 (49.1)	2 (100.0)		15 (60.0)	6 (75.0)	
Air bronchogram, *n* (%)						
Yes	48 (84.2)	1 (50.0)	0.313	15 (60.0)	4 (50.0)	0.695
No	9 (15.8)	1 (50.0)		10 (40.0)	4 (50.0)	

Preinvasive, AAH + AIS; Invasive, MIA + IA; GGO, ground‐glass opacity.

## Discussion

Surgical intervention remains the cornerstone in the treatment of lung cancer and is the only way to offer long‐term survival.^11^ It has been reported that five‐year‐survival in patients with AIS and MIA presenting with GGO nodules on chest CT could be 100% following surgical resection.^5^ However, in another study, the five‐year survival rates in stage IA1, IA2, and IA3 which presented in GGO nodules were 97.8%, 89.3%, and 88.5%, respectively.^12^, ^13^ Hence, the surgical timing for the GGO nodules should be considered at the MIA stage. Previous reports have revealed that pure GGO nodules could be diagnosed as IA ultimately after surgical resection^14^, ^15^ and IA could also be misdiagnosed as a clinically noninvasive tumor on a preoperative CT scan just according to the CT features.^16^ In this study, we found that the part‐solid GGO nodules were significantly different in the invasiveness compared to the pure GGO nodules. A solid component is a risk factor for invasiveness and could be considered as the surgical indication when physicians encounter a new diagnosed of GGO nodule. Previous studies have also mentioned that a solid component in GGO nodules was related to invasiveness.^17^, ^18^, ^19^ Furthermore, the correlation of the solid component diameter and invasiveness was significantly positive,^7^ and this concept was also announced in the report by Zhang *et al*. which revealed the most powerful factor to predict IA was the diameter of the solid component.^20^ Moreover, the patients with solid‐predominant nodules were at a greater risk of recurrence compared to those with GGO‐predominant nodules.^21^ Therefore, part‐solid GGO nodules should be regarded as having a higher risk of invasiveness and surgical intervention should be considered.

Tumor size of the GGO nodule was another critical risk factor of potential malignancy.^22^, ^23^ We also found that tumor size was a significant factor for the invasiveness. A cutoff diameter of 10 mm for preinvasive GGO nodules and 14 mm for invasive GGO nodules have been previously reported.^6^ Eguchi *et al*. also reported a cutoff diameter of 11 mm for evaluating the invasiveness in pure GGO nodules.^24^ Recently, Li *et al*. reported that the GGO nodules showed a tendency to be invasive adenocarcinoma if the tumor diameter was larger than 13.5 mm.^25^ Until now, there has been no definitive surgical policy for pure GGO nodules and generally, a pure GGO nodule larger than 1 cm should be considered indicative for surgery. Previous studies have also revealed that all the IA presented in pure GGO nodules were larger than 1 cm.^16^ Our study revealed that a nodule diameter larger than 1 cm was a significant factor to predict invasiveness in the part‐solid group.

Quantitative analysis of attenuation of the GGO nodule by histogram distribution has offered physicians a way to distinguish preinvasiveness from invasiveness and the report by Li *et al*. showed that the tumor volume and mean attenuation could be a predictive factor for the invasiveness.^26^ In the study by Zhao *et al*. mean CT value was chosen for distinguishing the preinvasiveness from the invasiveness.^27^ We also found a similar result in our study that there was a correlation between the tumor volume and invasiveness. The mean CT value did not reveal the significant difference in the present study, despite the values being similar in comparison with the data in the study by Zhao *et al*. However, maximal tumor attenuation was another significant factor for the invasiveness reported in our study and was compatible with the study by Li *et al*.^28^ Moreover, the mean attenuation of the solid component has been evaluated in a previous study and revealed a significant difference between preinvasiveness and invasiveness.^29^ Thus, tumor attenuation could be considered as a useful tool to evaluate invasiveness.

The morphology between preinvasive nodules and invasive nodules has also been previously reported and the smooth margin noted, mainly in preinvasive lesions.^6^ An irregular margin of the GGO nodules was positively associated with invasive lesions.^27^ In our study, we did not find a correlation between the nodular margin and invasiveness, and we infer that the small size of the invasive group may be the major cause. Bubble lucency of the GGO nodule was chosen as the CT feature in a previous study to distinguish the IA from AIS/MIA, although the result revealed no significant difference.^20^ In our study, we used bubble lucency to evaluate the invasiveness and the result indicated that this feature could not be considered as the predictive tool. An air bronchogram had been used to evaluate invasiveness although the efficacy in invasiveness prediction is still controversial. However, an air bronchogram was found to be a good prognostic factor in the study by Yoshino *et al*.^30^ but, in contrast, another study showed that the presence of an air bronchogram was more frequently noted in IA than in AIS.^31^ An air bronchogram was not a significant factor to predict invasiveness in our study. Hence, according to our results and those of other studies, morphology of the GGO nodules should not be regarded as a good predictor for invasiveness.

There were some limitations in our study. First, it was a single‐institution retrospective study and we only selected the patients with small GGO nodules (less than 2 cm) with a C/T ratio less than 0.25. Therefore, there may have been a bias in the population which could not be applied to other types of GGO nodules where the tumor size was greater than 2 cm or C/T ratio larger than 0.25. Second, the total sample size was small, especially in the invasive group. Further studies are required to re‐evaluate the invasiveness with an increase in the sample sizes of the invasive group. Third, the nodule margin was drawn freehand and thus there may have been slight personal error in tumor volume and CT value evaluation. In future, to reduce the possibility of personal error, further software upgrades are required in order to be able to evaluate the nodular margin more precisely, instead of relying upon manual margination. Nonetheless, this study still offers an important reference for evaluating the invasiveness of the small and low C/T ratio of GGO nodules to assist physicians in formulating a treatment strategy.

In conclusion, when GGO nodules are detected on a CT scan, the physician has to decide if the nodules are potentially invasive and whether surgical intervention should be performed at the MIA stage. The clinical features including tumor size, solid‐component, tumor volume and maximal tumor attenuation are significant factors in the prediction of invasiveness. In particular, unlike the NCCN guideline, we suggest that part‐solid GGO nodules with a diameter greater than 1 cm should be given surgical consideration due to their high possibility of invasiveness.

## Disclosure

No authors report any conflict of interest.
